# Accurate diagnosis of pulmonary inflammatory myofibroblastic tumor by imaging technology before operation: A case report

**DOI:** 10.1097/MD.0000000000034798

**Published:** 2023-09-01

**Authors:** Lv Sun, Yuhang Zhu, Cheng Chen, Jiajia Huang, Bangguo Li

**Affiliations:** a Department of Radiology, Affiliated Hospital of Zunyi Medical University, Medical Imaging Center of Guizhou Province, Huichuan District, Zunyi, Guizhou, P. R. China; b Department of Anesthesiology, Affiliated Hospital of Zunyi Medical University, Huichuan District, Zunyi, Guizhou, P. R. China; c Department of Thoracic Surgery, Affiliated Hospital of Zunyi Medical University, Huichuan District, Zunyi, Guizhou, P. R. China; d Department of Pathology, Zunyi Maternal and Child Health Care Hospital, Honghuagang District, Zunyi, Guizhou, P. R. China.

**Keywords:** pulmonary nodules, inflammatory myofibroblastic tumor, imaging diagnosis, case report

## Abstract

**Rationale:**

Pulmonary inflammatory myofibroblastic tumor (IMT) is a rare borderline tumor, which has the potential of malignant including invasion of surrounding tissues, distant metastasis and recurrence. However, the preoperative diagnosis is difficult and it can also be difficult to distinguish from malignancy in small tissue samples. Preoperative accurate diagnosis has important clinical significance for patients to choose treatment measures and improve the quality of rehabilitation. We was examined by computed tomography (CT) plain scan plus enhanced scan, magnetic resonance diffusion-weighted imaging (DWI) and apparent diffusion coefficient (ADC) imaging technology in an adult female, compared with lung cancer and pulmonary cryptococcus infection for diagnosis of pulmonary IMT.

**Patient concerns:**

A 32-year-old female patient was admitted to the hospital “physical examination revealed nodules in the right upper lung for 1 week”.

**Diagnoses:**

The patient was diagnosed with Pulmonary inflammatory myofibroblastic tumor.

**Interventions:**

Single-port thoracoscopic lobectomy was performed after multidisciplinary consultation.

**Outcomes:**

DWI and ADC improves the accuracy of preoperative diagnosis and well guides the formulation of treatment measures. The combined CT, DWI, and ADC magnetic resonance imaging technology has more important significance in the diagnosis and differential diagnosis of IMT and lung malignant tumors.

**Lessons:**

Although accurate preoperative diagnosis of pulmonary IMT is difficult. Chest CT examination combined with DWI and ADC imaging technology has high clinical significance for the diagnosis of IMT.

## 1. Introduction

The increase of health examination and the improvement of medical technology make the detection rate of pulmonary masses increase dramatically. The growth, evolution, nature and size of the masses determine the different of treatment methods. Benign pulmonary nodules can be treated conservatively, while malignant tumors need the combination of multiple treatment methods, mainly surgery. An expert consensus agreed that it is of great significance for the diagnosis and treatment of related diseases and the long-term health of patients, especially during the COVID-19 pandemic.^[1]^

Inflammatory myofibroblastic tumor (IMT) is a kind of soft tissue tumor that commonly occurs in children and young adults. “The WHO Classification of Soft Tissue and Bone Tumors,” prepared by the WHO Editorial Board of Cancer in 2020, classified IMT as an intermediate (occasionally metastatic) tumor.^[2]^ The lung, omentum, mesentery and other soft tissues are the most common sites of IMT, among which the lung accounts for about 1/3, most of which show local invasive growth, and about 5% of the total IMT may show distant metastasis.^[3,4]^ Surgical resection is the main treatment for tumors in situ, while targeted therapy is more relied on for distant metastasis and recurrent tumors.^[5]^ Since the overall incidence of pulmonary IMT is low and difficult to diagnose, Multidisciplinary imaging examination can provide effective diagnostic basis, which can provide effective guidance for the selection of surgical treatment before metastasis of this disease, and helpful to improve the long-term health quality of patients.

## 2. Patient information

A 32-year-old female patient was admitted to the hospital “physical examination revealed nodules in the right upper lung for 1 week.” she did not show any symptoms or signs of respiratory disease in the past 3 months. No previous history of respiratory diseases such as recurrent wheezing, cough, expectoration, hemoptysis, infection, etc, and no person from a high-risk area of COVID-19.

## 3. Clinical findings

The thorax was symmetrical. There were no wet or dry rales on auscultation. Lung function tests were normal.

## 4. Timeline (Fig. 1)

**Figure 1.**
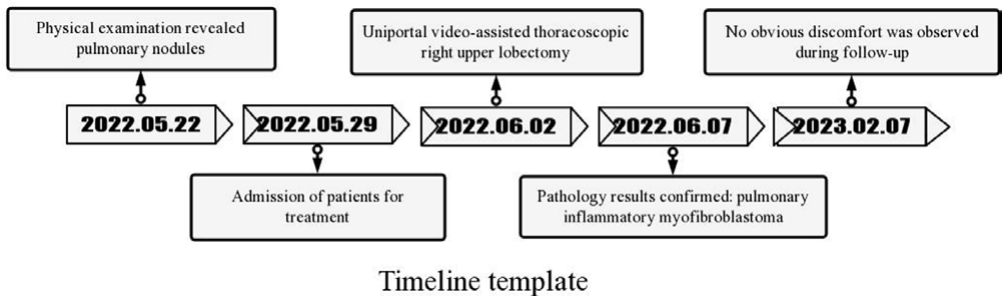
Timeline tamplate.

## 5. Diagnostic assessment

**Figure 2.**
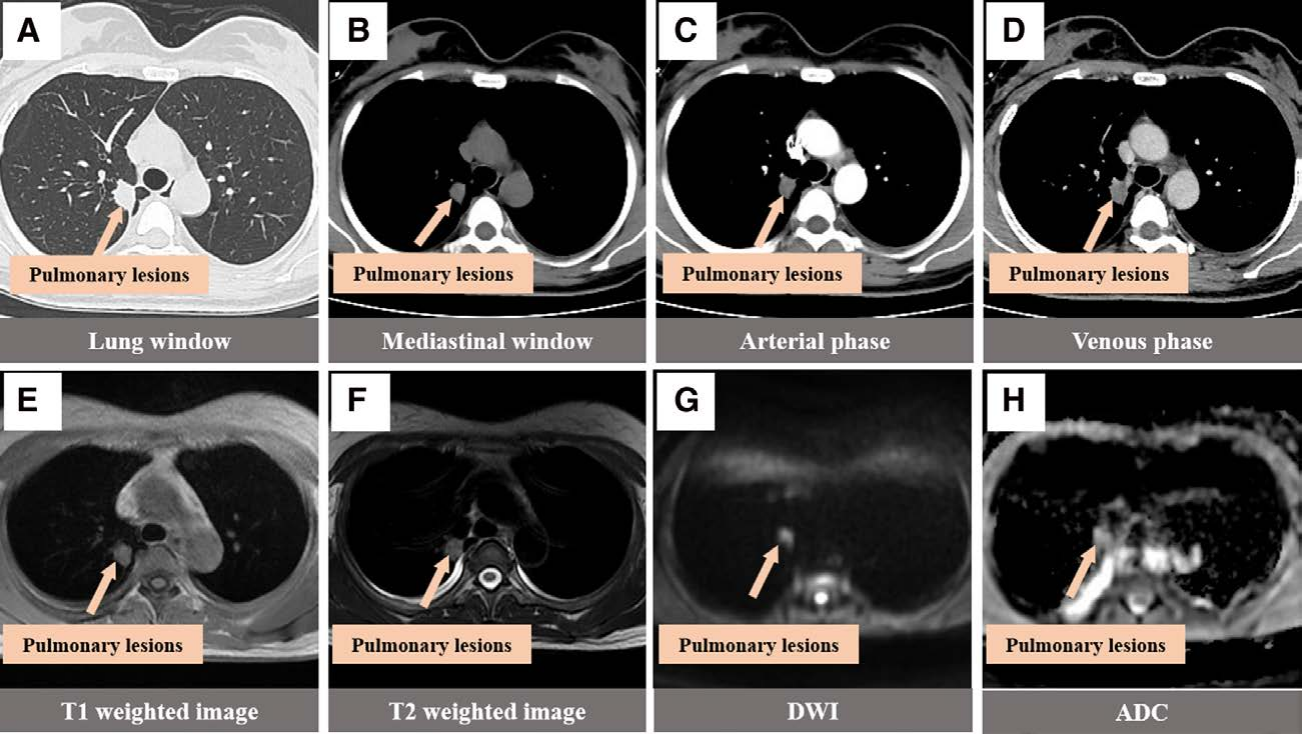
CT images (A–D) and MRI images (E–H) of the patient. (A, B) Pulmonary window and mediastinal window: oval soft tissue density nodules in the right upper lung, (C, D) enhanced arteriovenous phase: mild to moderate uneven delayed enhancement with slight thickening of adjacent pleura on enhanced scan. MRI images were injected with oval, T1 and slightly longer T2 signal nodules in the right upper lung of (E, F) with a small amount of pleural effusion, (G) nodules showed hyperintensity on DWI, and (H) ADC nodules showed hyperintensity. ADC = apparent diffusion coefficient, CT = computed tomography, DWI = diffusion-weighted imaging, MRI = magnetic resonance imaging.

Chest computed tomography (CT) in other hospital showed nodules in the upper lobe apex of the right lung, and sclerosing alveolar cell tumor and carcinoid were considered. Admission diagnosis: right upper lung mass. Chest CT examination was performed on the same day after admission (Fig. 2A–D), which showed soft tissue density nodules of about 23 × 16 × 19 mm^3^ in the right upper lung, with shallow lobulated edges and adjacent pleural traction depression, mild to moderate uneven enhancement on enhanced scanning, and small mediastinum and hilar lymph nodes. Imaging diagnosis: right upper lung nodules, inflammatory lesions are likely. To further determine the surgical plan, chest magnetic resonance imaging examination was performed the next day (Fig. 2E–H). The results showed T1 and slightly longer T2 signal nodules with a size of 23 × 16 × 19 mm3 in the right upper lung, with superficial lobulated edges, high intensity on diffusion-weighted imaging (DWI) and high intensity on apparent diffusion coefficient (ADC), adjacent pleural thickening, no enlargement of mediastinal and hilar lymph nodes, and a small amount of bilateral pleural effusion. Imaging diagnosis: Nodules in the right upper lung tend to be inflammatory lesions, and tumors were excluded by close follow-up; Bilateral small amount of pleural effusion. Blood test: WBC9.34 × 109/L, N 0.77%, L 0.12%.

## 6. Therapeutic intervention

**Figure 3.**
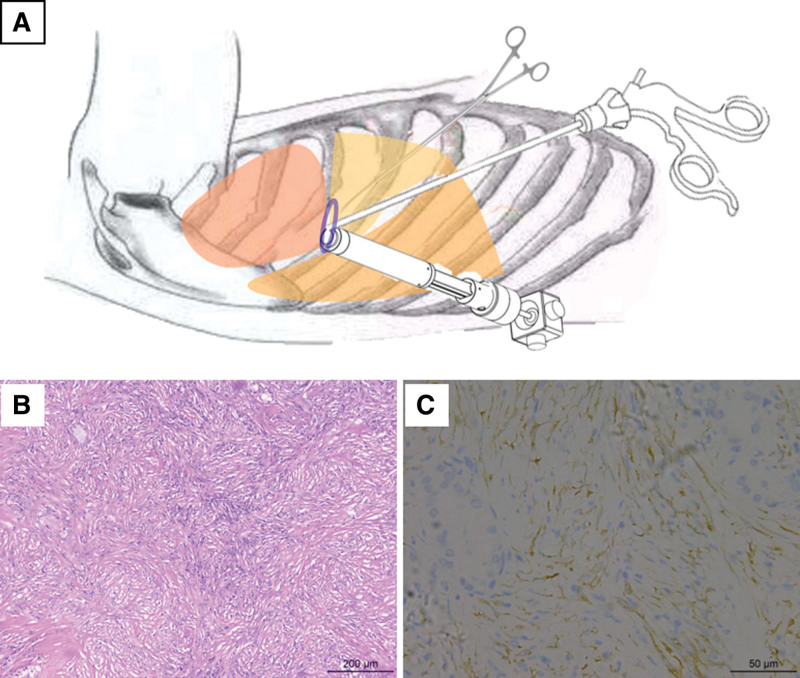
Schematic diagram of single-port thoracoscopic operation (A) and pathological examination results (B,C). A large number of spindle cell hyperplasia with lymphocyte and plasma cell infiltration, local lymphoid follicle formation, scattered alveolar epithelial cell hyperplasia, immunohistochemistry showed the expression of tumor cells: CK focal (+), ALK (+ +), SMA (+ +), Desmin (−), according to (−), CD34 (−), Ki67 (5%). The expression of CD138 (++), IgG (++), IgG4 (−) in interstitial plasma cells; Napsin-a (+) and TTF1(+) were expressed in alveolar epithelial cells.

The surgical plan was determined based on the opinions of the imaging department, the Department of pathology and the Department of anesthesiology. Single-port thoracoscopic lobectomy was performed (Fig. 3A). The operation process was smooth. After the operation, the endotracheal tube was removed and returned to the ward awake.

## 7. Follow-up and outcomes

Immunohistochemical results of postoperative paraffin sections showed a right upper pneumonic myofibroblastoma (Fig. 3B and C). The patient was discharged from hospital 1 week later. No special discomfort was observed during 8 months of follow-up.

## 8. Discussion

IMT is a rare tumor with systemic onset but unknown origin, especially occurring in the chest tissue, and the incidence of lung tumors is <1%.^[6]^ IMT is an intermediate tumor with a tendency to develop into malignant tumors. Pulmonary IMT may occur at all ages, and patients may present with asymptomatic symptoms, chronic cough, hemoptysis, and progressive dyspnea according to the disease evolution, tumor size and location.^[7–9]^ It is difficult to make a direct diagnosis before histopathological results are obtained. With the promotion of the development of multidisciplinary cooperation, it is of practical clinical significance to make a clear preoperative diagnosis for the choice of surgical methods, improve the treatment effect, and promote rapid recovery for such patients.

**Figure 4.**
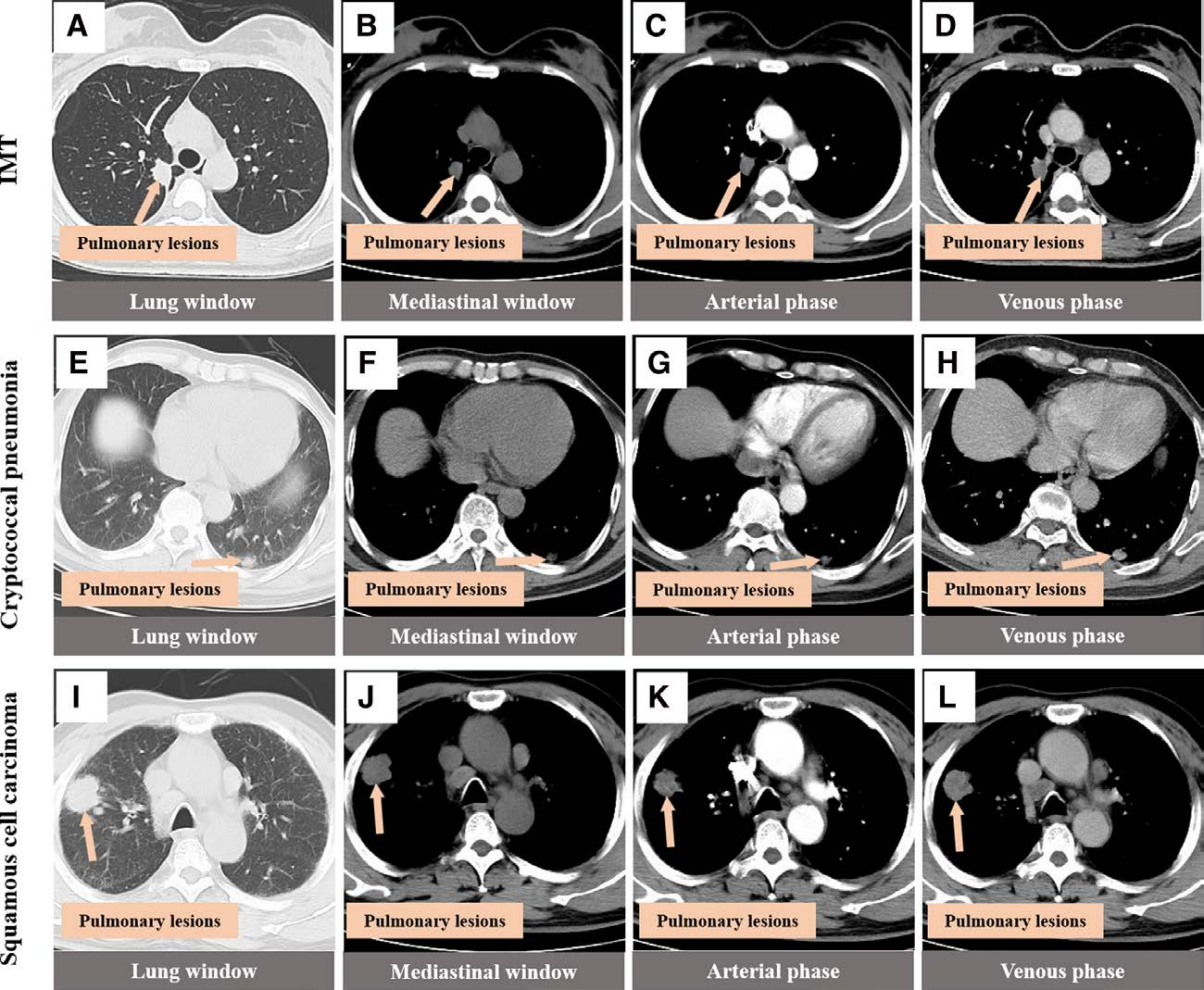
CT findings of three different lung masses note. (A–D) Oval soft tissue density nodules in the right upper lung, with shallow lobulated edges, mild to-moderate uneven delayed enhancement on enhanced scanning, and slight adjacent pleural thickening. (E–H) Oval soft tissue density nodules in the left lower lung with blurred boundary and obvious uniform enhancement on enhanced scan. (I–L) Circular soft tissue density nodules in the right upper lung with deep lobulated edges, marked uneven enhancement on contrast-enhanced scan with small areas without enhancement. CT = computed tomography.

**Figure 5.**
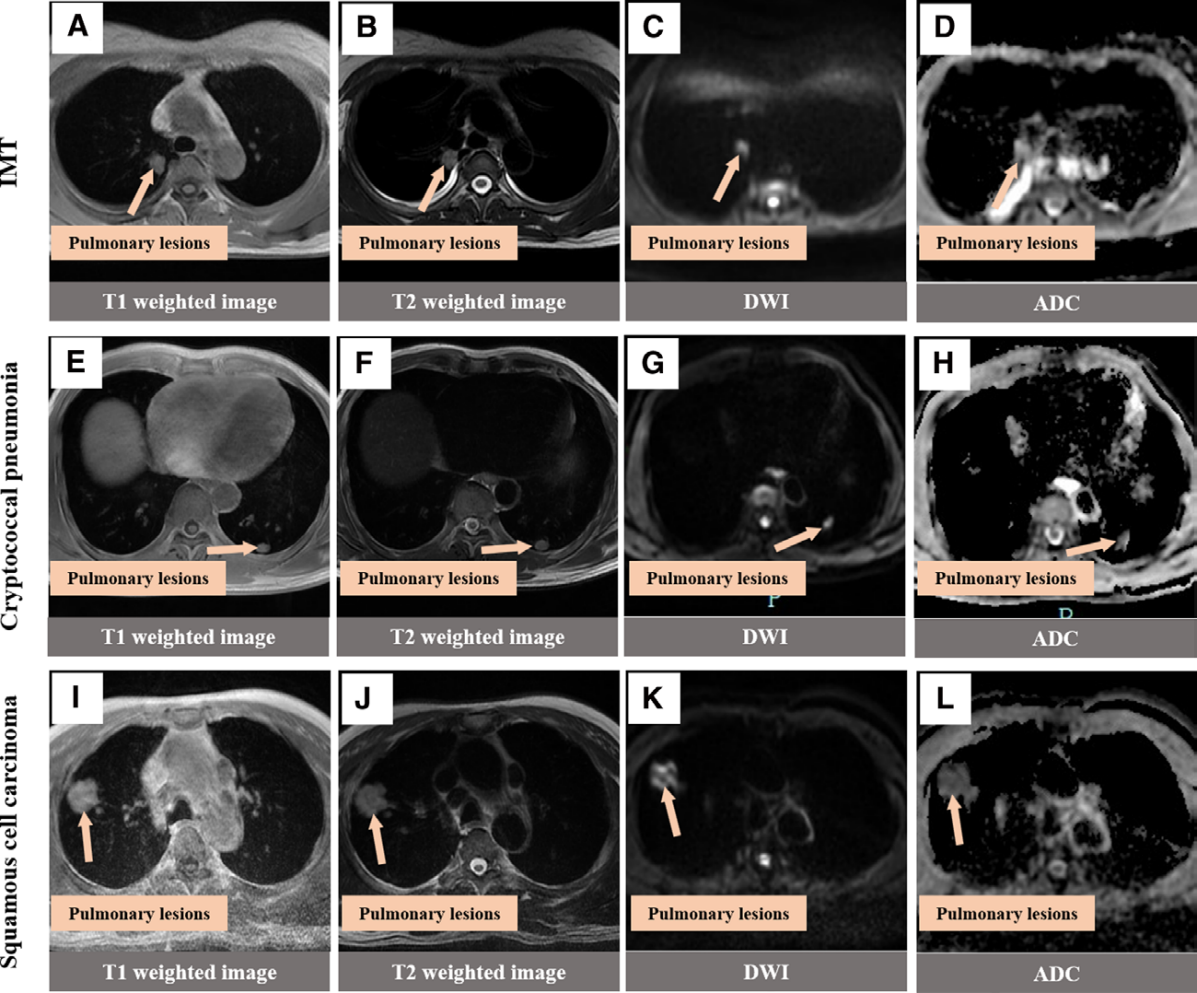
MRI findings of three different types of lung masses Note. (A–D) The T1 and T2 signals of oval shape in the right upper lung were slightly longer, with high signal on DWI and high signal on ADC. (E–H) In the left lower lung, the T1 signal was slightly longer than the T2 signal, the DWI showed a high signal, and the ADC showed a slightly high signal. (I–L) The right upper lung is round, equal to T1 and slightly longer T2 signal, high signal on DWI and low signal on ADC. ADC = apparent diffusion coefficient, DWI = diffusion-weighted imaging, MRI = magnetic resonance imaging.

Imaging combined with tissue puncture and pathological examination is an effective method to determine the nature of lung masses, but this method has risks and disadvantages such as small sample size, vascular injury, and tumor metastasis.^[10]^ In clinical practice, it is more common to rely solely on imaging to preliminarily define the nature of the mass and make diagnosis and treatment plans. In many imaging reports of IMT of the lung, CT examination results are almost all used, which usually suggest solitary nodules in the lung, with different shapes and usually no lymphadenopathy. Enhanced scanning is mainly characterized by different degrees of delayed enhancement,^[9,11,12]^ which is similar to the CT findings of this case. For now, MR manifestation of pulmonary inflammatory muscle fibroblastoma is given priority to with case reported, the patients were MR performance for T1 is a bit long T2 signals, such as no necrosis internal signals, do not agree with Naime reports,^[13]^ consideration may be related to this study small nodules, has not yet occurred necrosis and inflammatory muscle fibroblastoma image performance related diversification. Magnetic resonance DWI and ADC are of great significance for the differentiation of benign and malignant tumor lesions in multiple organs of the body, including the lung,^[14–17]^ and the ADC value of benign lung lesions is higher than that of malignant lung lesions.^[18]^ In this patient with IMT, DWI, and ADC sequence examination were added before operation, and the results showed that both DWI and ADC of the right upper lung nodules showed hyperintensity. In addition, CT and MR findings of pulmonary cryptococcal infection, pulmonary IMT and lung cancer were preliminarily compared in this study (Figs. 4 and 5). IMT showed shallow lobulation, the boundary of cryptococcal infection was blurred, and lung cancer showed deep lobulation. IMT showed mild to moderate uneven delayed enhancement, cryptococcal infection showed obvious uniform enhancement, and lung cancer showed obvious uneven enhancement. Magnetic resonance imaging of the 3 lesions showed that the nodules showed the same T1 signal and slightly longer T2 signal, and the DWI of lung cancer showed high signal and significantly reduced ADC signal, which was consistent with the report of Shen et al^[19]^ IMT and DWI of cryptococcal infection showed high signal intensity, and there was no significant difference between the signal intensity and lung cancer. ADC signal intensity of lung cancer was the lowest, while that of IMT was the highest. ADC signal intensity of pulmonary cryptococcal infection was higher than that of lung cancer and slightly lower than that of IMT. However, ADC signal intensity of IMT and cryptococcal infection was similar, so it was still difficult to distinguish them. In conclusion, the combined DWI and ADC imaging technology has certain value in the diagnosis and differential diagnosis of IMT and lung malignant tumors, and the differential diagnosis value of IMT and benign lung lesions needs to be further explored. The thoracic surgeon combined with the good suggestions given by the imaging, comprehensively analyzed the results and sought the opinions of the related experts in pathology and anesthesiology, designed the precise surgical plan and completed the operation with the surgical method of lung tumors with nonmalignant tendency.

## 9. Conclusion

After pulmonary nodules appear in children or young patients, chest CT examination combined with DWI and ADC imaging technology has high clinical significance for the diagnosis of IMT, which has reference value for doctors to choose conservative treatment and surgical treatment, and is helpful for the long-term health of patients.

## Author contributions

**Conceptualization:** Lv Sun.

**Investigation:** Yuhang Zhu, Cheng Chen, Jiajia Huang.

**Project administration:** Lv Sun.

**Resources:** Cheng Chen, Jiajia Huang, Bangguo Li.

**Supervision:** Bangguo Li.

**Visualization:** Yuhang Zhu.

**Writing – original draft:** Lv Sun.

**Writing – review & editing:** Lv Sun.

## References

[1] Mazzone PJ Gould MK Arenberg DA. Management of lung nodules and lung cancer screening during the COVID-19 pandemic: CHEST expert panel report. Chest. 2020;158:406–15. 32335067 10.1016/j.chest.2020.04.020 PMC7177089

[2] Mahajan P Casanova M Ferrari A. Inflammatory myofibroblastic tumor: molecular landscape, targeted therapeutics, and remaining challenges. Curr Probl Cancer. 2021;45:100768. 34244015 10.1016/j.currproblcancer.2021.100768

[3] Janik JS Janik JP Lovell MA. Recurrent inflammatory pseudotumors in children. J Pediatr Surg. 2003;38:1491–5. 14577073 10.1016/s0022-3468(03)00501-3

[4] Reinke DK. Meaningful engagement of the patient in rare cancer research: sarcoma as an exemplar. Curr Probl Cancer. 2021;45:100772. 34289946 10.1016/j.currproblcancer.2021.100772

[5] Karnak I Senocak ME Ciftci AO. Inflammatory myofibroblastic tumor in children: diagnosis and treatment. J Pediatr Surg. 2001;36:908–12. 11381424 10.1053/jpsu.2001.23970

[6] Ekinci GH Haciomeroglu O Sen AC. Inflammatory myofibroblastic tumor of the lung. J Coll Physicians Surg Pak. 2016;26:331–3. 27097710

[7] Yang F Zhang W Han C. A case of pulmonary inflammatory myofibroblastic tumor treated with bronchoscopic therapy plus lobectomy. J Cardiothorac Surg. 2021;16:144. 34039398 10.1186/s13019-021-01528-5 PMC8157757

[8] Ikeda T Nakano J Kushida Y. Multiple pulmonary inflammatory myofibroblastic tumor. Kyobu Geka. 2019;72:367–70. 31268035

[9] Braham Y Migaou A Njima M. Inflammatory myofibroblastic tumor of the lung: a rare entity. Respir Med Case Rep. 2020;31:101287. 33251105 10.1016/j.rmcr.2020.101287 PMC7683262

[10] Na Y Park S. Inflammatory myofibroblastic tumor of the pleura with adjacent chest wall invasion and metastasis to the kidney: a case report. J Med Case Rep. 2018;12:253. 30195334 10.1186/s13256-018-1796-7 PMC6129296

[11] Ufuk F Herek D Karabulut N. Inflammatory myofibroblastic tumor of the lung: unusual imaging findings of three cases. Pol J Radiol. 2015;80:479–82. 26568776 10.12659/PJR.894902 PMC4621161

[12] Li H He Y Wang B. Rare cases of pulmonary inflammatory myofibroblastic tumors in adult male patients: a case report. Transl Cancer Res. 2021;10:4274–80. 35116724 10.21037/tcr-21-1683 PMC8797295

[13] Naime S Bandarkar A Nino G. Pulmonary inflammatory myofibroblastic tumour misdiagnosed as a round pneumonia. BMJ Case Rep. 2018;2018:bcr2017224091. 10.1136/bcr-2017-224091 PMC5836705 29455182

[14] Santos MK Elias J Jr Mauad FM. Magnetic resonance imaging of the chest: current and new applications, with an emphasis on pulmonology. J Bras Pneumol. 2011;37:242–58. 21537662 10.1590/s1806-37132011000200016

[15] Baba S Isoda T Maruoka Y. Diagnostic and prognostic value of pretreatment SUV in 18F-FDG/PET in breast cancer: comparison with apparent diffusion coefficient from diffusion-weighted MR imaging. J Nucl Med. 2014;55:736–42. 24665089 10.2967/jnumed.113.129395

[16] Thörmer G Otto J Reiss-Zimmermann M. Diagnostic value of ADC in patients with prostate cancer: influence of the choice of b values. Eur Radiol. 2012;22:1820–8. 22527373 10.1007/s00330-012-2432-3

[17] Kuang F Ren J Zhong Q. The value of apparent diffusion coefficient in the assessment of cervical cancer. Eur Radiol. 2013;23:1050–8. 23179520 10.1007/s00330-012-2681-1

[18] Tondo F Saponaro A Stecco A. Role of diffusion-weighted imaging in the differential diagnosis of benign and malignant lesions of the chest-mediastinum. Radiol Med. 2011;116:720–33. 21293944 10.1007/s11547-011-0629-1

[19] Shen G Jia Z Deng H. Apparent diffusion coefficient values of diffusion-weighted imaging for distinguishing focal pulmonary lesions and characterizing the subtype of lung cancer: a meta-analysis. Eur Radiol. 2016;26:556–66. 26003791 10.1007/s00330-015-3840-y

